# CAMD-RTDETR: Real-Time Multi-Defect Detection Method for Tunnel Structures

**DOI:** 10.3390/s26134112

**Published:** 2026-06-29

**Authors:** Yunyun Hao, Xiangyang Xu

**Affiliations:** School of Rail Transportation, Soochow University, Suzhou 215006, China; 20244246005@stu.suda.edu.cn

**Keywords:** tunnel multi-defect detection, CAMD-RTDETR, deep learning, cross-attention feature mining, multi-scale feature pooling, decoding enhancement, real-time object detection

## Abstract

Intelligent tunnel defect detection is essential for structural safety and efficient operation and maintenance. However, manual inspection is inefficient, subjective, and risky, while existing deep learning methods often show unstable performance under practical conditions involving small targets, large-scale variations, and severe background interference, limiting their accuracy and real-time deployment on edge devices. To address these issues, this paper proposes CAMD-RTDETR, an end-to-end real-time multi-defect detection method based on RT-DETR. Cross-attention feature mining is introduced to enable bidirectional interaction between shallow spatial details and deep semantic information, enhancing the perception of weak-texture defects such as fine cracks. Multi-scale contextual pooling is designed to aggregate features from different receptive fields and improve the unified representation of cracks, seepage, and spalling with diverse morphologies. In addition, decoding enhancement and query optimization are incorporated to improve query updating and localization discrimination, thereby enhancing detection stability and boundary accuracy in complex tunnel scenes. Experiments on a field-collected tunnel defect dataset show that CAMD-RTDETR achieves an average inference latency of 15.855 ms per image and a processing speed of 63.06 FPS under the batch-size-1 testing setting. Compared with the baseline RT-DETR, Precision, Recall, mAP50, and mAP50-95 are improved by 6.3%, 13.5%, 14.7%, and 15.8%, respectively. Comparisons with seven representative detectors further demonstrate its superior accuracy and real-time performance, demonstrating its preliminary feasibility for edge-side inference and its potential for future integration into vehicle-mounted tunnel inspection systems.

## 1. Introduction

Tunnel engineering is one of the most important infrastructural foundations for ensuring urban transportation operation and promoting regional economic development. During long-term service, tunnels are frequently affected by multiple factors, such as groundwater infiltration, dynamic vehicular loads, and environmental erosion, which often lead to defects including cracks, water leakage, spalling, and voids. These defects not only reduce the durability and stability of tunnel structures but may also trigger serious accidents such as concrete falling, lining failure, and even collapse, thereby posing significant threats to traffic safety and public life and property [1,2]. Previous studies have shown that water leakage, cracks, and spalling account for more than 70% of lining defects in metro tunnels in China [3], highlighting the great practical significance of defect detection and assessment for the safe and healthy operation of tunnels.

Traditional tunnel defect detection mainly relied on manual inspection and simple image-processing methods, such as crack width measurement using calipers, threshold segmentation, and edge detection [4,5]. These approaches are not only inefficient and labor-intensive, but their detection results are also highly dependent on inspectors’ experience, making them strongly subjective and prone to missed and false detections [6,7]. In addition, the complex tunnel environment, characterized by insufficient lighting, surface contamination, and structural aging, further increases the difficulty of manual inspection [4]. Therefore, researchers have begun to explore more intelligent and automated detection methods.

In recent years, the development of computer vision and deep learning has provided new solutions for tunnel defect detection. Detection methods based on CNN, FCN, R-CNN, and YOLO series models have been widely applied to the identification of defects such as cracks, water leakage, and spalling, achieving promising results [6,8,9]. On this basis, researchers have continuously explored more efficient detection frameworks. For example, Feng et al. [10] proposed a two-stage deep learning framework to improve the recognition efficiency of multiple types of defects. Zhu et al. constructed MFF-YOLO through multi-scale feature fusion, effectively improving detection accuracy [11]. Fang et al. achieved real-time detection at 64 fps using YOLOv10, satisfying the requirements of online inspection [12]. Huang et al. proposed an intelligent detection method based on LiDAR point clouds and an improved YOLOv8 model for four common types of metro tunnel defects, achieving favorable engineering application performance [13]. Fang et al. proposed YOLO-Tunnel, an improved YOLOv5-based model, for internal lining defects such as cavities, voids, and insufficient compactness, achieving accurate detection with both high precision and real-time performance on ground-penetrating radar B-scan images [5]. Duan et al. addressed the low efficiency and high risk of manual defect detection for tunnel portal walls in plateau regions by proposing an intelligent detection method based on UAV images and an improved DeeplabV3+ model, enabling safe and efficient identification and quantification of multiple portal-wall defects [14].

In terms of detection framework optimization, the MB-YOLO model proposed by Lu [6] integrates mixed local channel attention (MLCA) and a bidirectional feature pyramid network (BiFPN), outperforming YOLOv3, YOLOv5, YOLOv9, YOLOv10, and other algorithms in both accuracy and speed. Zheng et al. combined coordinate attention with a Transformer to improve the detection capability for small defects [1]. Yao et al. proposed an ultra-high-resolution image defect detection framework suitable for deep learning models, significantly enhancing detection performance [15]. Juan et al. achieved lightweight detection through knowledge distillation [16], and Zhao et al. realized underground defect identification based on an improved YOLOv5 model and GPR data [17]. Wang et al. developed an improved YOLOv7 model integrating ConvMixer, BiFPN, and SimAM, enabling high-precision and rapid detection of apparent lining defects [18]. These studies have continuously achieved breakthroughs in model accuracy, speed, and lightweight design; however, their cross-scenario robustness under complex working conditions remains insufficient.

Meanwhile, automated and intelligent detection systems oriented toward practical applications have also emerged. The system developed by Li et al. can automatically identify cracks and water leakage [19]. Dang et al. achieved crack segmentation and parametric measurement using an improved U-Net [13], further promoting quantitative defect detection. Huang et al. effectively distinguished defects from bolt-hole interference using FCN [20]. To address the insufficient efficiency of perception, analysis, and decision-making in railway tunnel operation and maintenance, Zhao et al. proposed an overall framework for intelligent railway tunnel operation and maintenance based on digital twins, providing systematic support for defect detection, condition assessment, and auxiliary decision-making [21]. Gao et al. integrated FCN and Faster R-CNN to achieve joint detection of multiple defect types [22]. Zhou et al. improved the detection performance for complex surface defects by optimizing anchor boxes and feature fusion [23]. Guo et al. developed an intelligent monitoring system based on machine vision and zoom cameras, enabling all-weather real-time identification and monitoring of multiple types of defects [24].

In terms of detection performance, Carion et al. proposed the DETR model [25], which reformulated object detection as a global set prediction problem. By introducing the Transformer architecture and the Hungarian matching algorithm, DETR established an end-to-end detection paradigm without the need for traditional anchor boxes or non-maximum suppression. However, to address the slow training convergence and poor small-object detection performance of DETR, researchers have proposed various improved methods. Meng et al. proposed Conditional DETR, which accelerates model convergence by decoupling content and spatial information [26]. Li et al. introduced denoising training in DN-DETR to alleviate the instability of bipartite matching, further improving training efficiency and stability [27]. Zhu et al. enhanced the model’s detection capability for small and multi-scale objects by optimizing the feature extraction network and multi-scale interaction mechanism [28]. These studies have promoted the application and development of DETR in real-time object detection.

Although deep learning has achieved significant progress in the detection of tunnel defects such as cracks, water seepage, and spalling, existing methods still face notable limitations in complex tunnel scenarios. Tunnel defects exhibit substantial scale variation: fine cracks are typically narrow and weak in texture, whereas spalling and seepage regions often occupy large areas and present irregular shapes. As a result, existing attention mechanisms and feature extraction methods find it difficult to simultaneously preserve fine details of small targets and effectively capture large-scale defects, leading to missed detections of cracks and blurred defect boundaries in scenarios characterized by weak textures, low contrast, and multi-scale coexistence. Meanwhile, the presence of lining textures, damp marks, reflective water stains, uneven illumination, and background noise in tunnel environments significantly increases interference, making conventional feature fusion structures prone to feature aliasing under complex backgrounds and thereby resulting in false detections and category confusion. In addition, although DETR-based models offer the advantage of end-to-end detection, their query mechanisms are relatively sensitive to high-noise backgrounds and fine-grained targets, which can lead to unstable query matching, degraded localization accuracy, and limited convergence efficiency under complex working conditions. Furthermore, while some methods can improve detection accuracy, their model architectures are often complex and computationally expensive, making them unsuitable for real-time deployment in vehicle-mounted tunnel inspection systems and edge devices. To address the above issues, this paper proposes CAMD-RTDETR, a real-time detection model for multi-scale tunnel defects, including cracks, water seepage, and spalling. Built upon RT-DETR, the proposed model introduces targeted improvements in three key aspects: feature interaction, multi-scale modeling, and decoding optimization. First, a Cross-Attention Feature Mining Module (CAFM) is constructed to establish bidirectional interactions between shallow detailed features and deep semantic features, thereby enhancing cross-level representation capability for both fine cracks and large-scale defects. Second, a Multi-Scale Feature Pooling Module (MSFP) is designed to strengthen contextual modeling across different receptive fields through parallel pooling and an adaptive channel fusion mechanism, thereby improving the model’s perception and fusion performance for multi-scale defect targets. Third, a Decoder Enhancement and Query Optimization Module (DAE) is introduced to reinforce encoded features via channel attention, spatial attention, and multi-scale deformable attention, while progressively optimizing target query representations, thus improving detection stability and localization accuracy under conditions of complex illumination, damp-mark interference, and densely distributed defects. The proposed CAFM, MSFP, and DAE are not simple combinations of existing attention or multi-scale modules. Instead, they are task-oriented designs developed for tunnel defect detection, where fine cracks often exhibit weak textures, seepage and spalling defects show large-scale irregular regions, and complex tunnel backgrounds may disturb feature representation and query matching. Specifically, CAFM focuses on bidirectional cross-level interaction between shallow spatial details and deep semantic features; MSFP enhances lightweight multi-scale contextual representation within the same feature layer; and DAE jointly improves encoded feature saliency and query optimization during the decoding stage. These designs enable the proposed model to better address weak-texture targets, multi-scale defect morphology, and background interference in real tunnel scenes. Experimental results demonstrate that the proposed model achieves high detection accuracy while maintaining an average inference latency of 15.855 ms and a processing speed of 63.06 FPS under the batch-size-1 testing setting, thereby achieving a favorable balance between detection accuracy and real-time performance. The model can be directly deployed in vehicle-mounted tunnel inspection systems and edge industrial control devices, offering substantial engineering value for improving the efficiency of intelligent tunnel defect inspection and reducing the safety risks associated with manual inspection.

## 2. Network Structure

**Figure 1:**
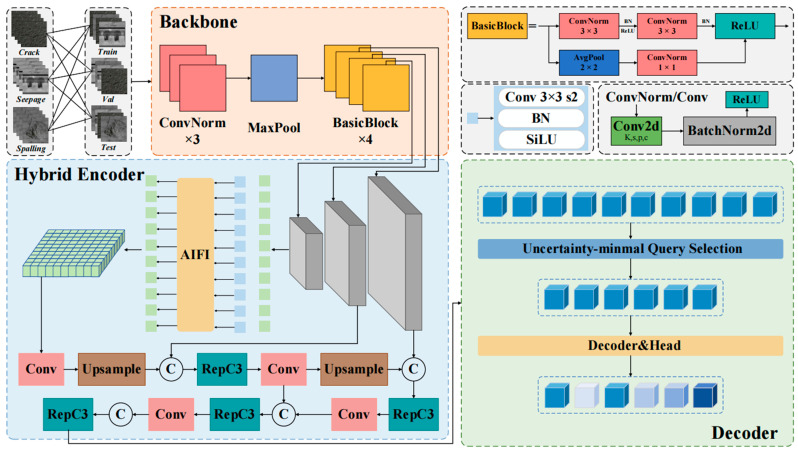
Network architecture of RT-DETR.

In this study, the Transformer-based RT-DETR framework is adopted as the core detection architecture, and its overall workflow is illustrated in Figure 1. RT-DETR consists of four main components: the Backbone, the Hybrid Encoder, Uncertainty-Minimal Query Selection, and the Decoder and Detection Head.

The backbone network consists of Conv Norm, Max Pool, and Basic Block structures, which are used to progressively extract low-level and high-level features from the input image. Specifically, the Basic Block is composed of convolution (Conv), normalization (Batch Norm), and activation functions such as ReLU and SiLU, thereby enhancing the feature representation capability. In the encoding stage, a Hybrid Encoder is introduced. By integrating the Adaptive Interaction Feature Integration (AIFI) module with multi-scale convolutional structures, including RepC3 and Upsample, the model achieves adaptive interaction and fusion of cross-scale features. This design enhances the modeling capability for multi-scale defect features while maintaining computational efficiency. In the query generation stage, an Uncertainty-Minimal Query Selection mechanism is adopted. By screening candidate regions, feature queries with high confidence and low uncertainty are preferentially selected, thereby improving the stability of object localization and classification. Finally, in the decoding and prediction stage, the Decoder & Head interacts with the selected queries with image features and progressively generates bounding-box and category prediction results. This process combines a multi-layer Transformer decoder with the detection head, enabling the model to maintain high small-object detection accuracy in complex tunnel scenarios.

Therefore, the overall architecture of RT-DETR not only balances the requirements of real-time performance and high detection accuracy but also effectively improves detection stability and generalization capability under complex backgrounds by introducing the Hybrid Encoder and the uncertainty-constrained query selection mechanism.

### 2.1. Cross-Attention Feature Mining Module

Tunnel defects such as cracks usually appear as fine, slender, and line-shaped structures with blurred boundaries. Their texture information is weak and their spatial continuity is poor; therefore, effective detailed features are mainly distributed in shallow high-resolution feature maps. In contrast, large-scale defects such as spalling and water seepage usually occupy larger areas, exhibit irregular morphologies, and have complex boundary variations, making them more dependent on deep semantic features for category discrimination and target recognition. During multi-level feature fusion, conventional feature pyramids mostly rely on simple upsampling, downsampling, or feature aggregation, which may cause shallow fine-grained information to be overwhelmed by deep semantic information. This results in insufficient responses to fine cracks and also limits the use of shallow spatial details for assisting boundary localization of large-scale defects.

To address these issues, this study introduces a cross-attention-based feature mining mechanism to establish bidirectional interactions between shallow spatial details and deep semantic information, enabling dynamic complementarity among features at different levels. On the one hand, the global semantic information contained in deep features can provide target-relevance guidance for shallow features, enhancing the network’s response to fine cracks, weak-texture defects, and low-contrast targets. On the other hand, the edge, texture, and positional information embedded in shallow features can supplement the spatial representation capability of deep features, thereby improving boundary perception and localization accuracy for large-scale irregular defects such as spalling and water seepage. Through this cross-level semantic guidance and detail feedback mechanism, the model can alleviate the imbalance in multi-scale defect feature representation without significantly increasing computational complexity, thereby improving the collaborative representation capability for both fine cracks and large-scale defects. Compared with SE and CBAM, which mainly perform channel-wise or spatial reweighting on a single input feature map, CAFM explicitly models the correlations between feature maps from different hierarchical levels. SE emphasizes global channel recalibration, CBAM further introduces spatial attention to enhance salient regions, and Non-local attention mainly captures long-range dependencies within a single feature map. In contrast, CAFM constructs a normalized channel correlation matrix between shallow high-resolution features and deep semantic features, enabling deep semantic information to guide the response of weak-texture cracks while allowing shallow edge and positional details to supplement the boundary localization of large-scale defects. Therefore, CAFM is more suitable for tunnel defect scenes where fine cracks, seepage regions, and spalling areas coexist with large-scale variations.

In CAFM, shallow and deep features are first aligned before cross-attention computation. Let the shallow input feature be denoted as $X_{s}\in R^{B\times C_{s}\times H_{s}\times W_{s}}$ and the deep input feature be denoted as $X_{d}\in R^{B\times C_{d}\times H_{d}\times W_{d}}$, where B represents the batch size, $C_{s}$ and $C_{d}$ denote the channel numbers of the shallow and deep features, and $H_{s}\times W_{s}$ and $H_{d}\times W_{d}$ denote their spatial resolutions, respectively. To enable cross-level feature interaction, 1×1 convolution is first used to unify the channel dimensions of the two features to C, and upsampling or downsampling is then applied to adjust them to the same spatial resolution H×W. Therefore, $X_{1}$ and $X_{2}$ in Equation (1) represent the aligned feature maps, both with the dimension of B×C×H×W.

Let the two input feature maps be defined as follows:

(1) $$X_{1},X_{2}\in R^{C\times H\times W}$$

where $X_{1},X_{2}$ denote the two input feature maps, and C, H and W represent the number of channels, height, and width, respectively.

First, channel attention is applied to the input features to obtain channel-weighted representations:

(2) $$F_{i}=CA(X_{i}),i=1,2$$

where CA(·) denotes the channel attention operation, and $F_{i}$ represents the output of the i-th input feature after channel-wise weighting.

Subsequently, the channel representations of the two features are normalized, and their channel similarity matrix is computed. After Softmax normalization, the corresponding correlation weight matrix is obtained as follows:

(3) $$S=\mathrm{Soft}\;\max({\overset{\wedge }{F}}_{1}\cdot \overset{\wedge }{F_{2}^{T}})$$

where *S* denotes the channel correlation weight matrix, and ${\hat{F}}_{1}$ and ${\hat{F}}_{2}$ represent the normalized channel representations, respectively.

On this basis, the correlation matrix is used to update the channel-weighted features, thereby achieving cross-channel attention fusion:

(4) $$O_{1}=S\cdot F_{1},\;O_{2}=S\cdot F_{2}$$

where $O_{1}$ and $O_{2}$ denote the updated output features, respectively, and “·” represents the matrix multiplication operation.

Finally, the two updated features are concatenated along the channel dimension to obtain the module output:

(5) $$F_{out}=Concat(O_{1},\;O_{2})$$

where $F_{out}$ denotes the final output feature of the CAFM module, and Concat(•) represents the channel-wise concatenation operation.

**Figure 2:**
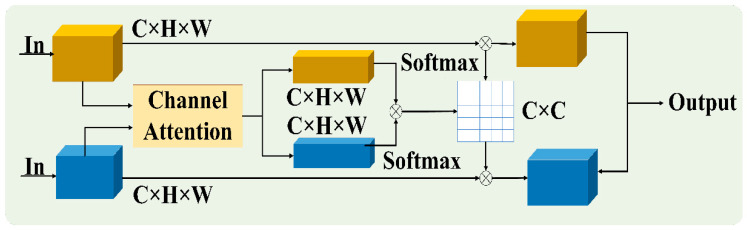
Structure of the CAFM Module.

During channel correlation calculation, the aligned features are first flattened along the spatial dimension. Let N=H×W; the two feature maps can then be reshaped into B×C×N. The normalized feature representations are multiplied to obtain the channel correlation matrix S, whose dimension is B×C×C. This matrix describes the channel-wise correlations between the two input features. The subsequent matrix multiplication can be understood as multiplying the correlation matrix with dimension B×C×C by the feature representation with dimension B×C×N, producing an output with dimension B×C×N. The output is then reshaped back to B×C×H×W, followed by channel-wise concatenation to obtain the CAFM output feature. In this way, CAFM maintains consistent output dimensions while achieving bidirectional interaction between shallow spatial details and deep semantic information. The specific structure of the module is shown in Figure 2.

Through the above design, the CAFM module is able to enhance the correlations among features at different hierarchical levels while maintaining high computational efficiency, thereby improving the discriminability and robustness of multi-scale feature fusion and providing more stable and effective feature representations for subsequent defect detection.

### 2.2. MSFP

Tunnel defects exhibit significant variations in spatial scale. Among them, cracks usually appear as slender, localized, and small-scale targets, whereas spalling and water seepage are often manifested as large-area regions with irregular shapes. A single-scale receptive field is insufficient to simultaneously capture the features of these markedly different targets, thereby limiting the model’s representation capability for multi-scale defects. To address this issue, this paper proposes a Multi-Scale Feature Pooling (MSFP) module, which enhances the model’s perception of defect targets at different scales by constructing multi-scale contextual information on the same feature layer.

The MSFP module first applies multiple average pooling operations with different receptive fields to the input feature map in parallel, so as to extract multi-scale contextual features. Subsequently, the multi-scale information is fused and reconstructed through feature concatenation, channel compression, normalization, and nonlinear activation. Finally, residual connections are introduced to preserve the original features, thereby alleviating information loss and improving the stability of gradient propagation. Without significantly increasing the computational burden, this structure effectively enhances the model’s joint representation capability for both fine-scale targets, such as cracks, and large-scale targets, such as spalling and water seepage. Different from SPP and ASPP, which mainly enlarge the receptive field through fixed pooling scales or atrous convolutions, MSFP constructs multi-scale contextual information through parallel average pooling within the same feature layer. Different from FPN and PANet, which rely on top-down or bottom-up cross-layer path aggregation, MSFP focuses on lightweight contextual enhancement inside a single feature level. By combining multi-branch pooling, feature concatenation, 1 × 1 channel compression, normalization, nonlinear activation, and residual learning, MSFP improves the unified representation of slender cracks and large-area seepage or spalling defects while keeping the computational overhead relatively low.

In MSFP, the input feature map is denoted as $X\in R^{B\times C\times H\times W}$, where B, C, H, and W represent the batch size, channel number, height, and width, respectively. This module adopts three average-pooling branches with kernel sizes of 3×3, 5×5, and 7×7 to extract contextual information under different receptive fields. To ensure that multi-scale features can be directly concatenated, all pooling branches use stride = 1 with appropriate padding, so that the output of each pooling branch remains B×C×H×W.

Let the input feature map be denoted as X. First, three two-dimensional average pooling operations with different kernel sizes are applied to it, yielding:

(6) $$P_{k}=AvgPool_{k}\left(X\right),\;k\in \left\{3,5,7\right\}$$

where $AvgPool_{k}\left(\cdot \right)$ denotes the two-dimensional average pooling operation with a kernel size of k×k, and $P_{k}$ represents the pooled feature map at the corresponding scale.

Then, the features at different scales are concatenated along the channel dimension:

(7) $$P=Concat\left(P_{3},P_{5},P_{7}\right)$$

where P denotes the concatenated multi-scale feature representation.

To reduce the number of channels and perform linear mapping, a 1 × 1 convolution is applied to transform the concatenated features:

(8) $$Q=Conv_{1\times 1}(P)$$

where $Conv_{1\times 1}(\cdot )$ denotes the 1 × 1 convolution operation, and Q represents the compressed feature representation.

Subsequently, batch normalization and the SiLU activation function are applied to the convolution output to enhance nonlinear representation capability:

(9) $$F=\mathrm{SiLU}\left(\mathrm{BN}\left(Q\right)\right)$$

where $\mathrm{BN}\left(\cdot \right)$ denotes the batch normalization operation, and $\mathrm{SiLU}\left(\cdot \right)$ denotes the activation function.

Finally, the enhanced features are added to the input features through a residual connection to obtain the final output of the MSFP module:

(10) Y=F+X

where Y denotes the final output feature of the MSFP module.

The outputs of the three pooling branches are concatenated along the channel dimension; therefore, the concatenated feature has the dimension of B×3C×H×W. A 1×1 convolution is then used to compress the channel dimension from 3C back to C, followed by Batch Normalization and the SiLU activation function for nonlinear transformation. Finally, the enhanced feature is added to the original input feature through a residual connection, and the output feature remains B×C×H×W. Therefore, MSFP preserves both the spatial resolution and the channel number of the input and output features, allowing it to be directly embedded into the feature enhancement process of RT-DETR.

**Figure 3:**
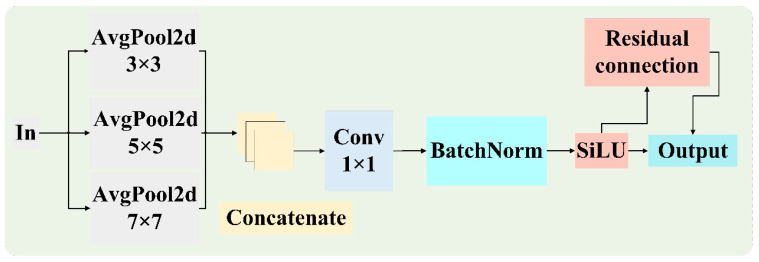
Structure of the MSFP Module.

Through the above design, the MSFP module can effectively integrate contextual information under different receptive fields within the same feature layer, thereby enhancing the network’s adaptability and robustness to multi-scale targets and improving the model’s detection capability in complex tunnel defect scenarios. The specific structure of the module is shown in Figure 3.

### 2.3. DAE

Tunnel images are typically characterized by insufficient illumination, surface contamination, damp-mark interference, and complex background textures. In particular, water seepage defects often exhibit blurred boundaries, while crack targets are small and easily confused with background noise. These factors make DETR-based models prone to unstable matching between queries and target regions during the decoding stage, resulting in a relatively high false detection rate and localization drift. To address these limitations, this paper designs a Decoder Augmentation and Query Optimization (DAE) module, which introduces a dual-attention mechanism and a query optimization strategy into the decoding stage, thereby improving the detection stability of the model in complex tunnel environments.

**Figure 4:**
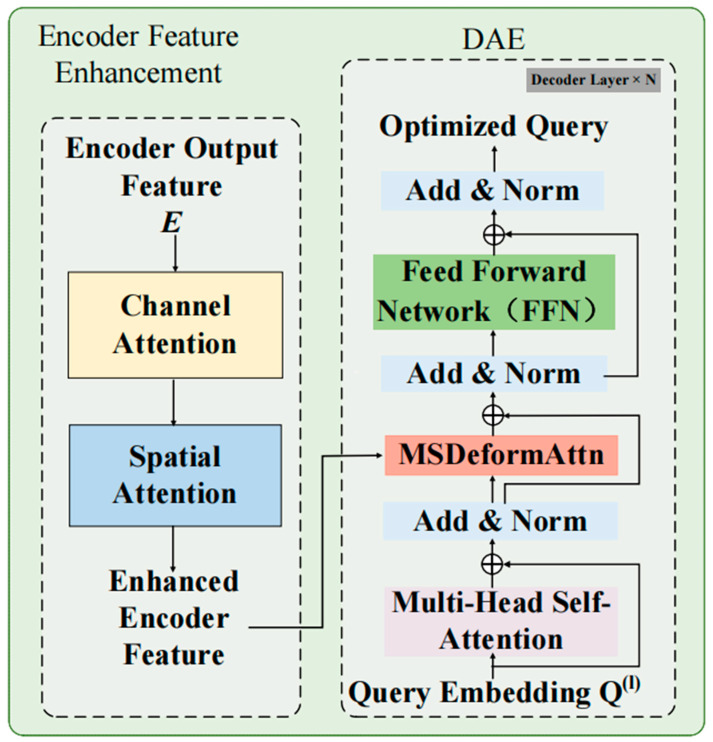
Structure of the DAE-Enhanced RT-DETR Decoder. Note: In Figure 4, SA and CA denote Spatial Attention and Channel Attention, respectively, while MSDeformAttn denotes Multi-Scale Deformable Attention.

Based on the encoder output features and the decoder input queries, the DAE module first employs a channel attention mechanism to reweight the important channels of the feature map, thereby highlighting key features relevant to defect targets. Subsequently, a spatial attention mechanism is introduced to further enhance the responses of potential target regions while suppressing interference caused by complex backgrounds. Finally, by integrating a multi-head attention mechanism, residual connections, and a feed-forward network, the target queries are progressively updated and optimized layer by layer, thereby enabling more accurate feature alignment and object localization. It should be noted that, in Figure 4, SA denotes Spatial Attention, and CA denotes Channel Attention; both are used to enhance the saliency of the encoder output features. The DAE module differs from Deformable DETR, DN-DETR, and existing RT-DETR improvements in its design objective. Deformable DETR mainly introduces multi-scale deformable attention to reduce the computational burden of Transformer-based detection and improve multi-scale object modeling. DN-DETR improves training convergence and matching stability through denoising training. RT-DETR focuses on efficient hybrid encoding and uncertainty-minimal query selection for real-time end-to-end detection. In contrast, DAE is specifically designed for tunnel images with low illumination, damp marks, lining textures, and strong background noise. Before query–feature interaction, DAE first enhances the saliency of encoded features using channel attention and spatial attention, and then updates the target queries through multi-scale deformable attention, residual connections, and feed-forward networks. Therefore, the main novelty of DAE lies in the joint enhancement of feature saliency, query matching stability, and localization accuracy under complex tunnel backgrounds.

In DAE, the encoder output feature is denoted as $E\in R^{B\times C\times H\times W}$, and the input query representation of the l-th decoder layer is denoted as $Q_{l}\in R^{B\times N_{q}\times C}$, where $N_{q}$ denotes the number of object queries and C denotes the query feature dimension, which is consistent with the channel dimension of the encoded feature. Before decoder interaction, the encoder output feature is first enhanced by channel attention and spatial attention to highlight potential defect regions and suppress background interference. For clarity, for a single feature level, the enhanced encoded feature can be flattened into a sequence representation with the dimension of B×HW×C. When multi-scale features are used, features at different scales are flattened according to the input format of MSDeformAttn and then participate in query–feature interaction.

Let the query feature input to the l-th decoder layer be denoted as $Q^{l}$, and let the encoder output feature be denoted as E. First, attention is applied along the channel dimension to obtain the weighted feature:

(11) $$E_{c}=\sigma (W_{2}\delta (W_{1}(GAP(E))))⊙E$$

where $GAP\left(\cdot \right)$ denotes global average pooling, $W_{1}$ and $W_{2}$ are learnable parameters, $\delta \left(\cdot \right)$ denotes the activation function, $\sigma \left(\cdot \right)$ denotes the Sigmoid function, and $\sigma \left(\cdot \right)$ represents channel-wise weighting.

Subsequently, a saliency weight map is generated along the spatial dimension:

(12) $$M_{s}=\sigma \left(f^{7\times 7}\left(\left[\mathrm{AvgPool}\left(E_{c}\right);\mathrm{MaxPool}\left(E_{c}\right)\right]\right)\right)$$

where $\left[\cdot ,\cdot \right]$ denotes the concatenation operation, $f^{7\times 7}\left(\cdot \right)$ denotes the 7 × 7 convolution, and $M_{s}$ represents the spatial attention weight map.

On this basis, the spatially enhanced feature representation is obtained as:

(13) $$E_{s}=M_{s}⊗E_{c}$$

Then, the query features and the enhanced encoder features are fed into the multi-head attention module for interactive updating:

(14) $$Q^{\left(l+1\right)}=\mathrm{MSDeformAttn}({\tilde{Q}}^{\left(l\right)},E_{s})$$

where MSDeformAttn(·) denotes the multi-scale deformable attention mechanism, $Q^{\left(l+1\right)}$ represents the updated query features, and ${\tilde{Q}}^{\left(l\right)}$ denotes the intermediate query representation obtained after the query features $Q^{\left(l\right)}$ are updated through multi-head self-attention and Add & Norm.

To improve the decoder’s adaptability to defect targets of different scales, this paper incorporates a Multi-Scale Deformable Attention (MSDeformAttn) mechanism into the query–feature interaction process. This mechanism enables adaptive sampling and aggregation at key locations over multi-scale feature maps, allowing the queries to focus more effectively on defect target regions under complex background conditions, thereby enhancing the feature alignment capability for defects of different scales, such as cracks, spalling, and water seepage. As illustrated in Figure 4, MSDeformAttn is employed to realize this cross-scale feature interaction process and, together with channel attention and spatial attention, forms the feature enhancement and query optimization mechanism in the decoding stage.

To further improve training stability and representation capability, residual connections and a feed-forward network are adopted in each decoding layer:

(15) $${\hat{Q}}^{l+1}=AddNorm\left(Q^{l+1}+\mathrm{FFN}\left(Q^{l+1}\right)\right)$$

where $\mathrm{FFN}\left(\cdot \right)$ denotes the feed-forward fully connected network, and ${\hat{Q}}^{l+1}$ represents the optimized query representation.

The channel attention weight has the dimension of B×C×1×1, which is used to recalibrate different channels of the encoded feature. The spatial attention weight has the dimension of B×1×H×W, which is used to enhance the spatial response of potential target regions. After channel and spatial attention enhancement, the encoded feature still maintains the dimension of B×C×H×W. The enhanced encoded feature is then converted into a sequence representation according to the decoder input format and interacts with the query feature $Q_{l}\in R^{B\times N_{q}\times C}$ through multi-scale deformable attention. After self-attention, multi-scale deformable attention, residual connections, normalization, and the feed-forward network, the output query representation remains $B\times N_{q}\times C$. Therefore, DAE progressively optimizes the object query representation without changing the query dimension, thereby improving query matching stability and localization accuracy in complex tunnel backgrounds.

Through the above design, the DAE module can actively suppress background noise during the decoding stage, enhance the saliency of defect target regions, and improve the stability of matching between queries and target features by integrating multi-scale deformable attention, thereby effectively improving detection accuracy and localization performance in complex environments.

### 2.4. Collaborative Mechanism

**Figure 5:**
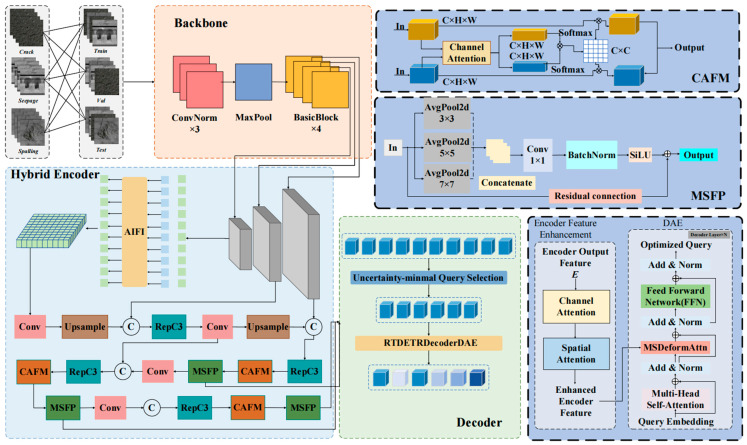
Overall Architecture of the Proposed CAMD-RTDETR Model.

To address the limitations of RT-DETR in tunnel defect detection tasks, including insufficient feature response to small-scale targets, limited representation capability for multi-scale targets, and inadequate decoding stability under complex background conditions, this paper develops an intelligent tunnel defect detection model, termed CAMD-RTDETR, whose overall architecture is illustrated in Figure 5.

The CAMD-RTDETR model mainly consists of a Backbone, a Hybrid Encoder, a Cross-Attention Feature Mining Module (CAFM), a Multi-Scale Feature Pooling Module (MSFP), a Decoder Augmentation and Query Optimization Module (DAE), and a Decoder. Compared with the baseline RT-DETR, the proposed model introduces dedicated structural modules in three key stages—feature interaction, context enhancement, and decoding optimization—to improve its detection capability for multiple tunnel defect targets while maintaining real-time performance.

Specifically, the CAFM is inserted between the multi-level features output by the backbone network and the input of the Hybrid Encoder. It is designed to perform cross-layer interaction and bidirectional guidance between shallow high-resolution detail features and deep high-semantic features, thereby enhancing the original multi-level feature fusion pathway. While the deep semantic information strengthens the responsiveness of shallow features to fine-scale defects such as cracks, the spatial details contained in shallow features can in turn complement the boundary localization capability of deep features. In this way, more discriminative cross-layer feature fusion is achieved before the features are fed into the Hybrid Encoder.

The MSFP is embedded into the feature enhancement process during the encoding stage and operates on the feature representations after cross-layer interaction. It acquires contextual information under different receptive fields through parallel pooling and reconstructs multi-scale information by means of channel compression and residual connections. MSFP primarily focuses on supplementing multi-scale contextual representation capability within a single feature level. This module enables the model to better accommodate the joint modeling requirements of small-scale targets, such as cracks, and large-scale defects, such as water seepage and spalling, while maintaining relatively low computational overhead.

The DAE is positioned between the encoder output and the decoder, and functions in the query optimization process during the decoding stage. Built upon the encoder output features and the decoder input queries, the DAE first enhances the saliency of the encoded features through channel attention and spatial attention prior to decoder interaction, and then progressively updates the query representations by integrating multi-scale deformable attention, residual connections, and a feed-forward network. In this way, the DAE enhances and reconstructs the original decoding mechanism of RT-DETR, thereby improving the stability of query-to-target matching and the accuracy of boundary localization under complex background conditions.

Based on the above structural design, CAMD-RTDETR establishes a coordinated mechanism consisting of front-end cross-layer feature interaction, middle-stage multi-scale contextual enhancement, and back-end decoding query optimization. For implementation, an input image batch is first processed by the backbone to extract multi-level features. CAFM aligns and interacts with shallow and deep features before they are fed into the Hybrid Encoder, while MSFP performs multi-scale contextual enhancement during encoding. The encoder outputs are then used for uncertainty-minimal query selection. In each decoder layer, the selected queries first undergo self-attention; meanwhile, the encoder features are enhanced by channel and spatial attention. The updated queries subsequently interact with the enhanced multi-scale encoder features through MSDeformAttn, followed by residual normalization and feed-forward transformation. Finally, the optimized queries are passed to the detection head to generate class predictions and bounding boxes. Specifically, CAFM is responsible for alleviating the information disconnection between shallow details and deep semantics; MSFP further improves the contextual representation capability of features for defect targets at different scales; and DAE suppresses complex background interference and enhances query stability during the decoding stage. These three modules are applied to different key components of RT-DETR, thereby preserving the real-time advantage of the original end-to-end detection framework while simultaneously improving the detection accuracy for multi-scale defects and complex scenarios. In summary, the proposed CAMD-RTDETR model effectively compensates for the limitations of the baseline RT-DETR in tunnel multi-defect detection through modular and stage-wise collaborative optimization. It provides an accuracy–efficiency-balanced solution for intelligent tunnel defect detection under complex working conditions.

In summary, CAFM, MSFP, and DAE improve RT-DETR from three different stages: cross-level feature interaction, single-level multi-scale contextual enhancement, and decoder query optimization. These modules are not simple replacements or direct stacking of existing attention and feature fusion structures. Instead, they form a task-oriented collaborative mechanism for tunnel defect detection, addressing weak textures, small targets, large-scale variations, irregular boundaries, and complex background interference. Through this stage-wise design, CAMD-RTDETR preserves the real-time end-to-end detection advantage of RT-DETR while improving feature representation, detection stability, and localization accuracy for multiple tunnel defect categories.

## 3. Experiments

### 3.1. Dataset Description

To address the limitations of existing public tunnel defect datasets, such as insufficient coverage of defect types, limited representativeness of engineering scenarios, and weak adaptability to complex environments, this study constructs a tunnel defect detection dataset containing three typical types of apparent defects—water seepage, cracks, and spalling—based on the practical requirements of tunnel operation and maintenance inspection. The dataset was collected from selected sections of metro shield tunnels in Xiamen and Tianjin, China. These two regions were selected because they represent different typical engineering geological conditions and provide diverse tunnel defect scenarios. Tunnel defect images were acquired by professional personnel using high-definition cameras during field inspection. The collected images include three types of apparent tunnel defects, namely seepage, cracks, and spalling. The original images were captured under real tunnel illumination conditions, including insufficient lighting, local reflection, non-uniform illumination, and interference from lining textures and auxiliary facilities. Therefore, the dataset reflects the complexity of practical tunnel inspection environments.

For the Xiamen region, the geological conditions are relatively complex and are mainly characterized by upper-soft and lower-hard composite strata, spherical weathering of granite, boulders, and water-rich fault zones. These conditions can easily induce uneven ground settlement, sudden changes in shield posture, and surrounding rock instability, which are important causes of tunnel seepage. As a result, seepage defects may occur at structural weak positions such as segment dislocation, opened joints, and damaged concrete areas. In addition, high-pressure groundwater seepage and chloride ion erosion in the marine environment may further aggravate seepage deterioration.

For the Tianjin region, the geological conditions are mainly characterized by thick muddy soft soil, a high groundwater level, and silty-fine sand layers. These conditions can easily lead to longitudinal tunnel settlement, segment deformation, and stratum liquefaction, which are also important causes of tunnel seepage. Consequently, seepage defects may occur at weak structural positions such as opened segment joints and deformed bolt holes. The rheological behavior of soft soil and long-term high hydrostatic pressure may further intensify the development of seepage defects.

The constructed dataset prominently reflects the characteristics of complex scenarios, multi-scale targets, and strongly interfering backgrounds. During data acquisition, real engineering conditions at tunnel sites were fully considered, ensuring that the sample distribution closely approximates the actual inspection environment during tunnel operation.

Unlike data acquired under laboratory conditions, where the background is relatively simple and imaging conditions are ideal, real tunnel images commonly suffer from insufficient illumination, local strong reflections, shadow occlusion, complex lining surface textures, obvious interference from background facilities, and substantial variations in shooting angles and distances. Therefore, they exhibit prominent characteristics of complex engineering scenarios. Meanwhile, water seepage, cracks, and spalling differ considerably in morphological structure, scale distribution, and spatial location. These defects include both small targets that are fine, slender, and characterized by blurred edges, as well as large-area targets with irregular boundaries, presenting typical characteristics of multi-category and multi-scale coexistence. Together, these factors increase the difficulty of defect detection and impose higher requirements on the model’s multi-scale feature extraction capability, complex background suppression capability, and cross-category discrimination capability.

Among them, cracks usually appear as slender, discontinuous linear targets with small widths, and are easily affected by lining textures, construction traces, and illumination variations. Spalling is mostly manifested as blocky, flaky, or irregular regions with a wide range of scale variations. Water seepage often appears as large-area diffusive regions or irregular areas distributed along joints, and can be easily confused with stains, water marks, and background color differences. To enhance the dataset’s representational capability for real operation and maintenance environments, complex samples involving low illumination, non-uniform lighting, local blurring, noise contamination, structural-joint interference, occlusion by auxiliary components, and confusion with texture-similar objects were retained as much as possible during dataset construction, rather than excessively filtering the original images. This strategy helps improve the model’s anti-interference capability and engineering applicability after training.

**Figure 6:**
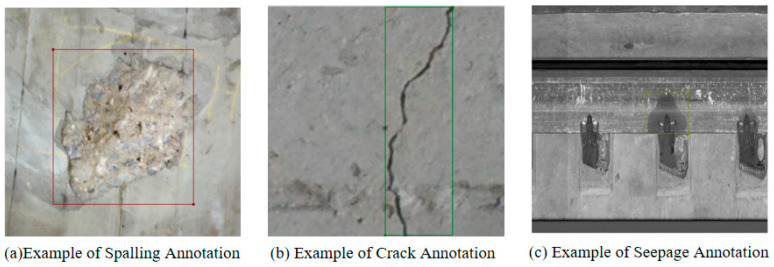
Schematic diagram of defect image annotation.

On this basis, the dataset used in this study contains three typical types of tunnel surface defects: spalling, cracks, and seepage. During the data preparation stage, a total of 2044 images were collected and annotated, including 432 images of spalling, 701 images of cracks, and 911 images of seepage. All images were annotated by the author using the LabelMe tool (version 4.5.13), and the defect regions were marked with rectangular bounding boxes, as shown in Figure 6. The annotation followed unified labeling criteria: cracks were labeled according to visible linear damaged regions, seepage was labeled according to visible wet areas, water stains, or diffusion regions, and spalling was labeled according to visible concrete surface peeling or damaged regions. For samples with ambiguous crack or seepage boundaries, the bounding boxes were determined based on the visually identifiable defect extent. After annotation, all labels were checked for category consistency, bounding-box rationality, and COCO-format conversion correctness. Overall, this dataset is characterized by strong engineering authenticity, diverse complex scenarios, pronounced multi-scale features, and evident co-occurrence of multiple defect types. Therefore, it can not only support single-defect detection studies, but it is also more suitable for the joint recognition of multi-category and multi-scale tunnel defects under complex environmental conditions.

During data annotation, spalling defects were labeled with red bounding boxes, crack defects with green bounding boxes, and seepage defects with yellow bounding boxes. This annotation strategy clearly reflects the differences among different defect types in terms of spatial location and morphological characteristics. Subsequently, the scattered JSON annotation files were uniformly converted into the COCO format, generating standardized images, annotations, and category structures. The dataset was then divided into training and testing sets at a ratio of 8:2 using stratified sampling. After cross-checking the category-wise statistics, the training set contained 1633 images, including 703 seepage images, 570 crack images, and 360 spalling images, while the testing set contained 411 images, including 208 seepage images, 131 crack images, and 72 spalling images. This strategy ensures that the proportions of the three defect categories remain consistent across the two subsets.

**Figure 7:**
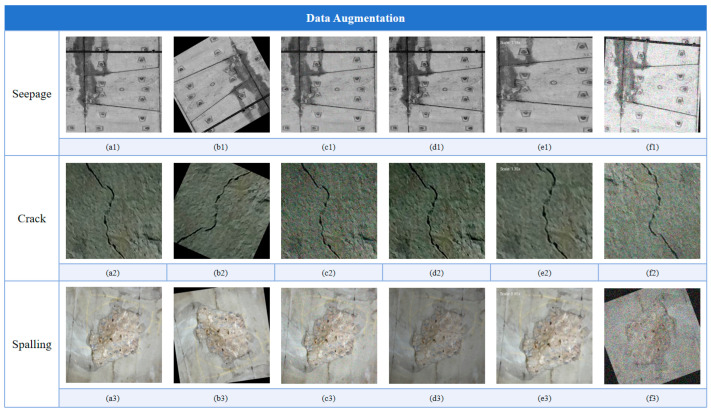
Schematic diagram of data augmentation for different tunnel defects. (**a1**–**f1**) seepage, (**a2**–**f2**) crack, and (**a3**–**f3**) spalling. In each group, (**a**–**f**) represent the original image, geometric transformation, noise disturbance, brightness adjustment, scale variation, and random enhancement, respectively.

The field environment of tunnel engineering is complex, and the collected images are often affected by uneven illumination, varying viewpoints, noise interference, and differences in defect scales. To ensure the generalization capability of the model under these variable conditions, data augmentation strategies were introduced into the training set in this study. As shown in Figure 7, the augmentation strategies include: (1) geometric transformation, which simulates different shooting angles and distances; (2) noise interference, which simulates low-light environments or sensor noise; (3) brightness and contrast adjustment, which simulates different illumination conditions inside tunnels; and (4) scale transformation, which ensures the scale invariance of the model in detecting defects of different sizes. By systematically increasing sample diversity in terms of morphological structure, texture characteristics, and illumination conditions, only the training set was augmented. After augmentation, the number of training images increased from 1633 to 2860, including 1271 seepage images, 988 crack images, and 601 spalling images, thereby providing the model with a training environment that closely approximates real engineering conditions.

**Figure 8:**
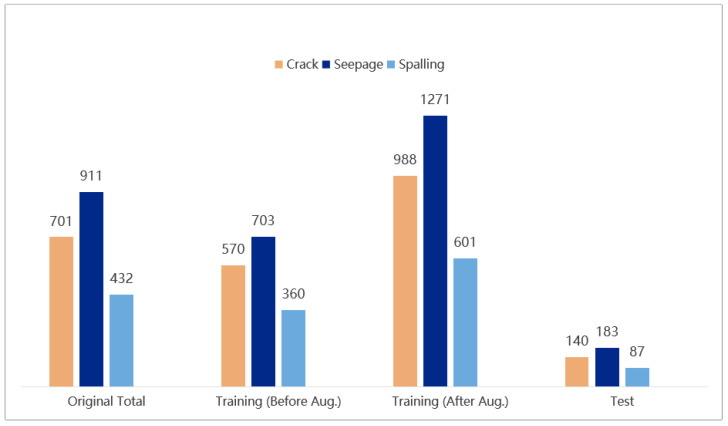
Comparison of Tunnel Disease Sample Quantities Before and After Data Augmentation.

Figure 8 presents the sample quantity distribution of the tunnel defect dataset at different stages. The horizontal axis represents the dataset partitioning stages, including the original total dataset, the training set before data augmentation, the training set after data augmentation, and the testing set. The vertical axis indicates the corresponding number of samples at each stage. The three polylines in the figure correspond to the three defect categories. Specifically, the blue line represents seepage, the orange line represents cracks, and the green line represents spalling. It can be observed that, after data augmentation, the number of samples in each defect category increased significantly. Specifically, the number of seepage samples in the training set increased from 703 to 1271, crack samples from 570 to 988, and spalling samples from 360 to 601. Therefore, the augmented training set contained 2860 images in total. The testing set was not augmented and contained 411 images, including 208 seepage images, 131 crack images, and 72 spalling images. These revised statistics are consistent with the category-wise breakdown shown in Figure 8.

### 3.2. Experimental Setup

In terms of experimental configuration, RT-DETR was adopted as the baseline detection framework, and the RT-DETR-L pretrained weights were used as the initialization parameters. Training and validation were conducted on the self-constructed tunnel defect dataset. The desktop-side training and evaluation environment was configured with Python 3.10.18 and PyTorch 2.7.1, and the hardware platform was an NVIDIA GeForce RTX 4060 GPU with 8 GB of memory. In addition, to further evaluate the edge-side deployment potential of the proposed method, TensorRT-based inference tests were conducted on an NVIDIA edge computing platform. During edge-side testing, different input resolutions were evaluated, and both pure TensorRT inference latency and folder-level end-to-end processing latency were recorded. During training, the input image size was uniformly set to 640 × 640, the batch size was set to 4, and the total number of training epochs was set to 500. The early stopping strategy was disabled to ensure sufficient model convergence. The SGD optimizer was employed, with an initial learning rate of 0.01, a final learning rate factor (lrf) of 0.01, and a momentum coefficient of 0.937. The learning rate scheduling strategy adopted is cosine annealing. For data augmentation, considering the large-scale variations, complex morphologies, and obvious low-light interference of tunnel defect targets, multiple augmentation strategies were applied during training, including Mosaic augmentation. Mosaic augmentation was disabled in the last 50 epochs to improve the model’s adaptability to the distribution of real images. Meanwhile, the random rotation angle was set to 0.0, the translation coefficient to 0.1, the scaling coefficient to 0.5, and the vertical flipping probability to 0.0. In addition, the number of data loading workers was set to 0. It should be noted that the default weight decay parameter, confidence threshold, and IoU threshold of the framework were used in all experiments. To ensure the fairness of the comparative experiments, both the baseline model and the proposed model were trained and tested under the same hardware environment, dataset partitioning strategy, and training parameter settings.

In the edge-side evaluation, TRT Latency denotes the average inference time of the TensorRT engine only, excluding image loading, preprocessing, postprocessing, and visualization. Folder Latency denotes the average processing time when continuously detecting images from a folder, including image loading, preprocessing, TensorRT inference, and postprocessing, but excluding visualization display. The corresponding FPS values are calculated from the average latency under each testing mode.

Edge-side deployment experiments were conducted on an NVIDIA Jetson Orin Nano. The platform integrates an Ampere-architecture GPU with CUDA cores and Tensor Cores, together with a multi-core ARM CPU, and is suitable for low-power deep-learning inference. The proposed CAMD-RTDETR model was converted and accelerated using TensorRT(NVIDIA TensorRT 10.7.0), and all edge-side latency tests were performed with a batch size of 1. TRT Latency denotes the execution time of the TensorRT inference engine only. Folder Latency denotes the sequential processing time of images in a folder, including image loading, preprocessing, TensorRT inference, and postprocessing. Thus, the folder-level test represents continuous offline image processing rather than a complete vehicle-mounted continuous inspection workflow.

### 3.3. Performance Evaluation Metrics

To comprehensively evaluate the performance of the detection model, this study adopts Precision, Recall, and the F1-score as evaluation metrics and further analyzes their variation trends in conjunction with different confidence thresholds.

(a) F1–Confidence Curve

The F1-score, which jointly considers Precision and Recall, is an important metric for evaluating the overall performance of the model. Its calculation is given in Equation (16):

(16) $$F1=\frac{2\times \mathrm{Precision}\times \mathrm{Recall}}{\mathrm{Precision}+\mathrm{Recall}}$$

(b) Precision–Confidence Curve

The Precision–Confidence curve reflects the reliability of the model outputs as the confidence threshold varies. The calculation of Precision is given in Equation (17):

(17) $$\mathrm{Precision}=\frac{TP}{TP+FP}$$

(c) Precision–Recall Curve

The Precision–Recall curve is an important comprehensive metric for evaluating the performance of a detection model. The calculation of Recall is given in Equation (18):

(18) $$\mathrm{Recall}=\frac{TP}{TP+FN}$$

(d) Recall–Confidence Curve

The Recall–Confidence curve reflects the recall performance of the model under different confidence thresholds. Its calculation is given in Equation (19):

(19) $$\mathrm{Recall}\left(\theta \right)=\frac{TP(\theta )}{TP(\theta )+FN(\theta )}$$

where TP denotes the number of defect targets correctly detected, FP denotes the number of defect targets incorrectly detected, and FN denotes the number of defect targets that were not detected. Precision, denoted as Precision (P), and Recall, denoted as Recall (R), are used to evaluate the detection performance, while the $F_{1}$ is the harmonic mean of Precision and Recall and serves as a comprehensive indicator of model performance. The symbol θ represents the confidence threshold, and the corresponding recall is denoted as Recall (θ); that is, the proportion of defect targets correctly detected under a given confidence threshold.

## 4. Experimental Analysis

### 4.1. Module Comparison

#### 4.1.1. Attention Mechanisms

Tunnel defect images contain not only fine linear targets such as cracks, but also relatively large regional defects such as seepage and spalling, exhibiting significant scale variation and complex backgrounds. A single channel-attention or spatial attention mechanism is insufficient to simultaneously capture both detailed and semantic information, while global self-attention is prone to interference when dealing with fine cracks. To address this issue, this study designs a Cross-Attention Feature Mining Module (CAFM), which enhances multi-scale feature representation through interactive fusion between shallow detailed features and deep semantic features. In object detection tasks, attention mechanisms can effectively improve the model’s capability to represent critical features. To verify the effectiveness of the proposed CAFM module, four representative attention mechanisms were selected for comparative experiments within the constructed RT-DETR framework, namely SE (Squeeze-and-Excitation) [29], CBAM (Convolutional Block Attention Module) [30], Non-local (Non-local Neural Network) [31], and Deformable Attention [32]. These comparisons were conducted to validate the effectiveness of the proposed module for tunnel defect detection. All modules were trained and tested under the same experimental environment and parameter settings, and the results are presented in Table 1.

In terms of box precision, the CAFM module achieved a value of 0.856, which is higher than those of SE (0.826), CBAM (0.810), and Non-local (0.839), and is also close to that of Deformable Attention (0.837). In terms of recall, CAFM achieved 0.631, which is lower than SE (0.656) and CBAM (0.639), but higher than Non-local (0.609) and essentially comparable to Deformable Attention (0.630). For the mAP@50 metric, CAFM achieved 0.752, outperforming all four methods. In terms of mAP@50–95, CAFM achieved 0.453, which is higher than SE (0.421), Non-local (0.437), and Deformable Attention (0.431), and is also close to CBAM (0.450).

Overall, CAFM demonstrates outstanding performance in key metrics such as box precision, mAP@50, and mAP@50–95, thereby verifying the effectiveness of the proposed interactive cross-feature-layer attention mechanism in complex tunnel defect scenarios.

#### 4.1.2. Multi-Scale Feature Pooling

Tunnel defects exhibit pronounced scale variation in images: cracks are typically slender and small in scale, seepage often appears in patch-like distributions, whereas spalling is commonly manifested as large-area block-like defects. Such diverse characteristics require the detection model to attend not only to subtle small targets, but also to large-scale defects, thereby imposing higher demands on multi-scale feature representation. Therefore, how to effectively enhance multi-scale feature modeling while maintaining real-time performance has become a critical issue in tunnel defect detection. To verify the effectiveness of the proposed MSFP module, comparative experiments were conducted on the same baseline model against several representative multi-scale feature modeling methods, including SPP (Spatial Pyramid Pooling) [33], ASPP (Atrous Spatial Pyramid Pooling) [34], FPN (Feature Pyramid Network) [35], and PANet (Path Aggregation Network) [36]. The experimental results are presented in Table 2.

The comparative results of MSFP and other multi-scale feature processing methods are presented in Table 3.

In terms of box precision, MSFP achieved 0.784, which is equal to FPN (0.784), slightly lower than SPP (0.788), but higher than ASPP (0.752) and PANet (0.732). In terms of recall, MSFP reached 0.632, which is higher than SPP (0.620), ASPP (0.617), and FPN (0.609), but slightly lower than PANet (0.637). For the mAP@50 metric, MSFP achieved 0.727, which is the best among the five methods, outperforming SPP (0.689), ASPP (0.723), FPN (0.690), and PANet (0.701). In terms of mAP@50–95, MSFP reached 0.469, which is significantly higher than SPP (0.408), ASPP (0.443), FPN (0.418), and PANet (0.414), demonstrating stronger overall detection capability.

Overall, while maintaining relatively high precision and recall, MSFP achieved the best performance on the two key metrics, mAP@50 and mAP@50–95, indicating that it can model the multi-scale characteristics of tunnel defects more effectively and has clear advantages in jointly detecting fine cracks as well as large-scale defects such as spalling and seepage.

#### 4.1.3. Decoder Augmentation and Query Optimization (DAE)

Tunnel defect detection scenarios are often accompanied by complex backgrounds and noise interference. Fine cracks are easily overwhelmed by noise, while patch-like seepage and block-like spalling frequently exhibit blurred or adhered boundaries. These characteristics make the query mechanism of DETR-based models prone to unstable matching during the decoding stage, thereby affecting localization and classification accuracy. To address this issue, this paper proposes a Decoder Augmentation and Query Optimization (DAE) mechanism to improve the model’s detection stability under complex working conditions. To verify the effectiveness of the proposed DAE mechanism, comparative experiments were conducted under a unified experimental setting against several representative DETR-based improvement methods, including Conditional DETR [30], which is based on conditional cross-attention; DAB-DETR [37], which employs dynamic anchor box queries; DN-DETR [31], which introduces denoising training; and Two-Stage Deformable DETR [36], which adopts a two-stage proposal refinement strategy. The experimental results are presented in Table 4.

The results in Table 4 indicate that different modules contribute differently to different evaluation metrics. The DAE module mainly improves the recall capability and decoding stability of the model. The MSFP module contributes more significantly to multi-scale contextual representation and achieves the highest mAP50-95 among the single-module variants. The CAFM module is more effective in enhancing feature discrimination and achieves the highest Precision among all ablation configurations. These results suggest that the proposed modules have different functional emphases rather than producing uniform improvements across all metrics.

In terms of box precision, DAE achieved 0.825, which is lower than DAB-DETR (0.859), but higher than Conditional DETR (0.797), and is also close to DN-DETR (0.832) and Two-Stage Deformable DETR (0.838). In terms of recall, DAE achieved 0.666, which is higher than DAB-DETR (0.624), DN-DETR (0.632), and Two-Stage Deformable DETR (0.618), and is essentially comparable to Conditional DETR (0.659). For the mAP@50 metric, DAE achieved 0.726, which is identical to DN-DETR and superior to the other three methods. In terms of mAP@50–95, DAE achieved 0.447, which is slightly lower than DN-DETR (0.449) but significantly higher than DAB-DETR (0.406), Conditional DETR (0.422), and Two-Stage Deformable DETR (0.408).

Overall, while maintaining relatively high box precision, DAE improves recall and detection accuracy under multiple IoU thresholds, achieves the best performance on mAP@50, and yields near-best results on mAP@50–95.

### 4.2. Ablation Study

To further analyze the independent contribution and synergistic effects of each proposed module, systematic ablation experiments were conducted based on the baseline RT-DETR framework. The experimental design included the individual integration of the three modules, namely CAFM, MSFP, and DAE, as well as different combinations of these modules, resulting in a total of seven comparative schemes. All experiments were performed under the same dataset and training parameter settings to ensure the comparability of the results. Four commonly used detection metrics, namely Precision, Recall, mAP50, and mAP50-95, were adopted for evaluation. These metrics were used to measure the model’s localization accuracy, detection capability, and overall detection performance under different IoU thresholds, respectively. The experimental results are presented in Table 5.

As shown in Table 5, Basic denotes the baseline RT-DETR model, while Config. 1, Config. 2, and Config. 3 correspond to the single-module variants with DAE, MSFP, and CAFM, respectively. Compared with Basic, Config. 1 increases Precision from 0.789 to 0.825, Recall from 0.601 to 0.666, mAP50 from 0.666 to 0.726, and mAP50-95 from 0.399 to 0.447, indicating that DAE improves decoding stability and query optimization. Config. 2 mainly enhances multi-scale contextual modeling, increasing mAP50-95 from 0.399 to 0.469. Config. 3 achieves the highest Precision among the single-module variants, increasing Precision from 0.789 to 0.856 and mAP50 from 0.666 to 0.752, demonstrating the effectiveness of CAFM in cross-layer feature interaction and detail representation.

For the combination experiments in Table 5, Config. 4, Config. 5, and Config. 6 correspond to the pairwise combinations of the proposed modules, while Config. 7 represents the complete CAMD-RTDETR model. The results indicate that the interactions among CAFM, MSFP, and DAE are nonlinear rather than simply additive. CAFM alone achieves the highest Precision of 0.856, whereas MSFP alone obtains the highest mAP50-95 of 0.469. In comparison, the complete model achieves the highest Recall and mAP50, while maintaining competitive Precision and mAP50-95. This suggests that the individual modules emphasize different aspects of feature representation and may partially overlap or compete when combined. Therefore, the main advantage of the complete CAMD-RTDETR lies in its balanced performance across multiple metrics rather than superiority in every individual metric. Since the present ablation results are based on single training runs under the same experimental settings, future work will conduct repeated experiments with different random seeds and report the mean and standard deviation to further evaluate statistical robustness. Specifically, Precision, Recall, mAP50, and mAP50-95 reach 0.839, 0.682, 0.764, and 0.462, respectively, corresponding to improvements of 6.3%, 13.5%, 14.7%, and 15.8% over the baseline RT-DETR. Although the final model does not obtain the highest value for every single metric, it achieves the highest Recall and mAP50 while maintaining competitive Precision and mAP50-95, demonstrating a favorable trade-off among localization accuracy, defect recall capability, and overall detection performance. These results indicate that the proposed modules contribute differently to different performance dimensions: CAFM mainly focuses on enhancing cross-layer feature interaction and detailed feature representation; MSFP primarily improves multi-scale contextual modeling; and DAE contributes to query optimization and decoding stability under complex backgrounds. The pairwise combinations do not produce linearly accumulated gains across all metrics, indicating that the interactions among CAFM, MSFP, and DAE are nonlinear. This may be because different modules emphasize different aspects of feature representation: CAFM enhances cross-level feature interaction and discriminative detail representation, MSFP strengthens multi-scale contextual modeling, and DAE improves query optimization and decoding stability. When these modules are combined, their effects may partially overlap or compete in specific metrics, leading to metric-dependent performance variations. Finally, when all three modules are introduced simultaneously, CAMD-RTDETR achieves the best comprehensive balance among the evaluated configurations, with Precision, Recall, mAP50, and mAP50-95 reaching 0.839, 0.682, 0.764, and 0.462, respectively. It should be noted that CAFM alone achieves a higher Precision of 0.856, and MSFP alone achieves a higher mAP50-95 of 0.469. Therefore, the advantage of CAMD-RTDETR lies not in being optimal for every individual metric, but in achieving stronger overall detection performance and better balance among different evaluation criteria. Compared with the baseline model, these values represent absolute improvements of 0.050, 0.081, 0.098, and 0.063, respectively. These results demonstrate that the collaborative design of front-end cross-layer feature interaction, middle-stage multi-scale contextual enhancement, and back-end decoding query optimization improves the overall detection capability of the model. Meanwhile, the ablation results also show that the contributions of different modules are metric-dependent, and their combinations involve nonlinear interactions rather than simple additive gains. Therefore, the proposed CAMD-RTDETR should be interpreted as a balanced and comprehensive optimization scheme for tunnel multi-defect detection, rather than a configuration that achieves the best value for every individual metric.

### 4.3. Comparison with Other Models

To comprehensively evaluate the performance of the proposed CAMD-RTDETR model, several representative object detection methods were selected for comparison. The comparative models include the two-stage detector Faster R-CNN, one-stage detectors including SSD, YOLOv5, and YOLOv8, as well as EfficientDet, RetinaNet, CenterNet, and the baseline RT-DETR, which are based on different architectural designs. The comparative experiments were conducted from two perspectives: detection accuracy and runtime efficiency. The evaluation metrics for detection accuracy included Precision, Recall, mAP50, and mAP50-95, while runtime efficiency was assessed by measuring the preprocessing time, postprocessing time, and overall inference latency. To ensure a controlled comparison, all models were trained and evaluated using the same training and testing splits and an input resolution of 640 × 640. A batch size of 4 and 500 training epochs were used, where supported, and early stopping was disabled. Publicly available pretrained weights and the specific model scales and backbones listed in Table 6 were adopted. No exhaustive model-specific hyperparameter search was performed on the tunnel defect dataset. Common settings were used when they were compatible across frameworks; otherwise, the officially recommended configuration of each implementation was retained. During testing, the confidence threshold was set to 0.25, and an IoU threshold of 0.50 was used where applicable. Therefore, the comparison follows a common data and evaluation protocol rather than identical architecture-specific optimization. The implementation details of all comparative models are summarized in Table 6.

All comparative models were trained on the same training set and evaluated on the same testing set. During training, the same image resolution of 640 × 640 and the same total number of epochs were used to ensure comparable optimization conditions. For data augmentation, geometric transformation, scaling, brightness adjustment, noise disturbance, and Mosaic augmentation were adopted, where supported by the corresponding framework. Mosaic augmentation was disabled in the last 50 epochs to reduce the distribution gap between augmented images and real tunnel images.

**Figure 9:**
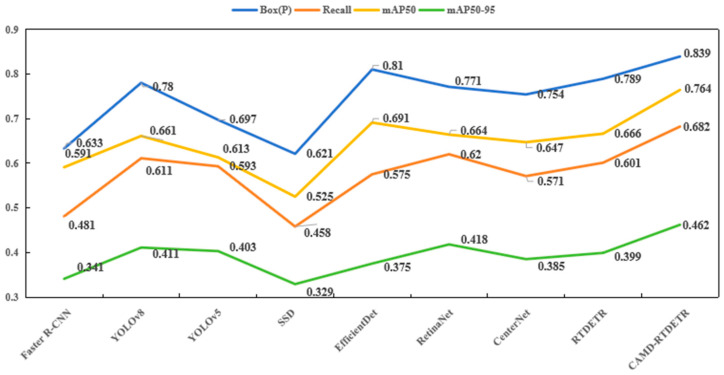
Comparison of Detection Performance Metrics among Different Models.

Figure 9 illustrates the performance of each model in terms of the four accuracy metrics.

From the results, the traditional Faster R-CNN and SSD exhibit relatively low overall performance in terms of mAP@50 and mAP@50–95. YOLOv5 and YOLOv8 achieve better precision and recall, but their adaptability to small targets and complex backgrounds remains limited. RT-DETR shows stable overall accuracy, whereas the proposed CAMD-RTDETR achieves the best performance across all four metrics, with a Precision of 0.839, a Recall of 0.764, an mAP@50 of 0.764, and an mAP@50–95 of 0.462. These results indicate that the introduced CAFM, MSFP, and DAE modules can effectively enhance the model’s capabilities in feature representation, multi-scale modeling, and decoder interaction.

**Figure 10:**
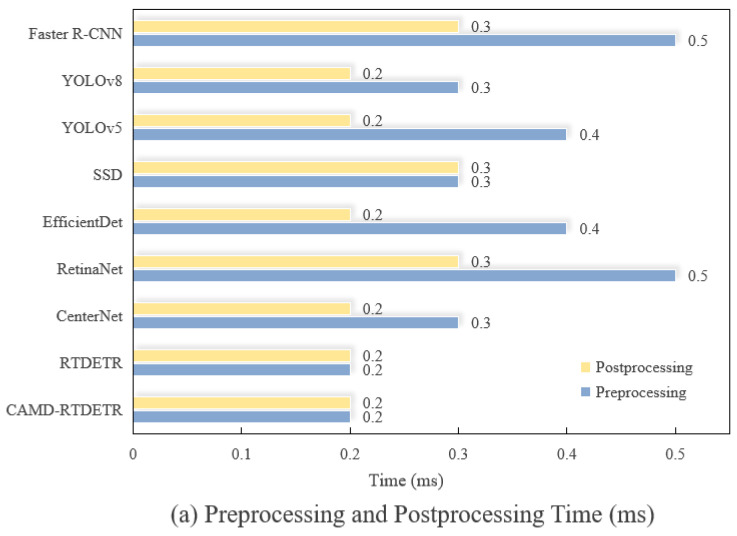

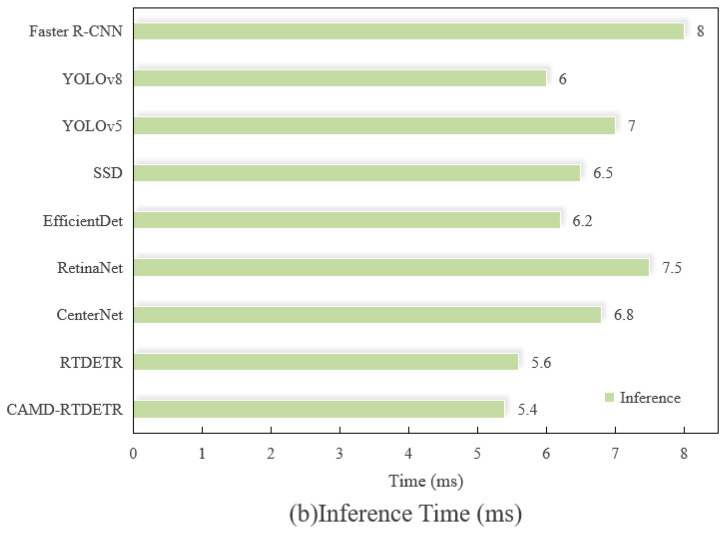
Comparison of the computational efficiency of different detection models: (**a**) preprocessing and postprocessing times; (**b**) inference latency.

In terms of computational efficiency, the experiments evaluated the models from three aspects: preprocessing time, postprocessing time, and inference latency. The results are shown in Figure 10. Specifically, Figure 10a presents the preprocessing and postprocessing times of each model, while Figure 10b illustrates the overall inference time.

In terms of runtime efficiency, Figure 10 compares the preprocessing and postprocessing time of different detection models. Since the original inference-time subfigure involved a different timing scope, it has been removed to avoid ambiguity. The standardized inference latency and FPS of CAMD-RTDETR are reported in Table 6. Under the batch-size-1 setting, CAMD-RTDETR achieves an average GPU-synchronized forward inference latency of 15.855 ms and a processing speed of 63.06 FPS.

According to the unified runtime evaluation results, CAMD-RTDETR achieves an inference latency of 15.855 ms and a processing speed of 63.06 FPS under the batch-size-1 setting. These results are consistent with the standardized runtime statistics reported in Table 5 and indicate that the proposed model maintains favorable real-time performance while improving detection accuracy.

To further evaluate the differences in computational complexity and runtime efficiency among different structural versions, this study systematically compares the baseline RT-DETR, the model variants incorporating CAFM, MSFP, and DAE, and the final CAMD-RTDETR model in terms of the number of parameters (Params), floating-point operations (FLOPs), inference latency under different batch sizes, frame rate, and peak memory usage. In Table 7, Latency@BS*n* denotes the average GPU-synchronized forward inference time required by the model to process one image batch under the batch-size-n setting, measured in milliseconds, where *n* = 1, 2, or 4. FPS@BS*n* denotes the corresponding image processing throughput under the same batch-size setting, measured in frames/s, and is calculated as follows:

$$FPS@BSn=\frac{n\times 1000}{Latency@BSn}$$

where BS*n* denotes the testing setting with batch size = n; n denotes the number of input images in a single batch; Latency@BS*n* denotes the average batch inference latency under the corresponding batch-size setting; FPS@BS*n* denotes the corresponding processing speed; and 1000 is used to convert milliseconds into seconds.

As shown in Table 7, the baseline RT-DETR has 20.08 M parameters and 58.2 G FLOPs. Under the condition of batch size = 1, its inference latency is 16.758 ms, corresponding to an FPS of 59.67. After incorporating the CAFM module, the number of parameters increases to 21.24 M, and the FLOPs increase to 64.5 G. The inference latency rises to 17.262 ms, while the FPS decreases to 57.92, indicating that enhanced cross-layer feature interaction introduces additional computational overhead while improving feature representation capability. For the MSFP version, the number of parameters further increases to 23.76 M, whereas the FLOPs decrease to 24.4 G. Under batch size = 1, the inference latency is 16.759 ms, and the FPS is 59.66, suggesting that this structure reconstructs the overall computational pathway while increasing the parameter scale. The DAE version contains 20.69 M parameters and 59.2 G FLOPs, with an inference latency of 17.736 ms and an FPS of 57.85, indicating that structural enhancement in the decoding stage has a certain impact on model complexity and inference efficiency. Finally, the proposed CAMD-RTDETR has 19.30 M parameters and 57.8 G FLOPs. Under the standardized batch-size-1 setting, CAMD-RTDETR achieves an average inference latency of 15.855 ms and a processing speed of 63.06 FPS. Under the batch-size-4 setting, the batch inference latency is 40.987 ms, corresponding to a throughput of 97.59 FPS. Overall, CAMD-RTDETR improves detection performance while maintaining favorable inference efficiency, demonstrating that the proposed method achieves a good balance between accuracy and real-time performance. It should be noted that different structural versions involve coupled adjustments in the detection head configuration and certain network connection strategies. Therefore, the results in Table 5 reflect the overall differences in complexity and runtime efficiency among different structural versions, rather than the pure incremental overhead introduced by a single module. In addition, the runtime values reported in the figure and Table 5 have been unified under the same batch-size-1 setting. Specifically, the inference latency denotes the average GPU-synchronized forward inference time of the model, while FPS denotes the corresponding image processing throughput. This unified definition ensures the consistency and comparability of runtime evaluation results.

**Figure 11:**
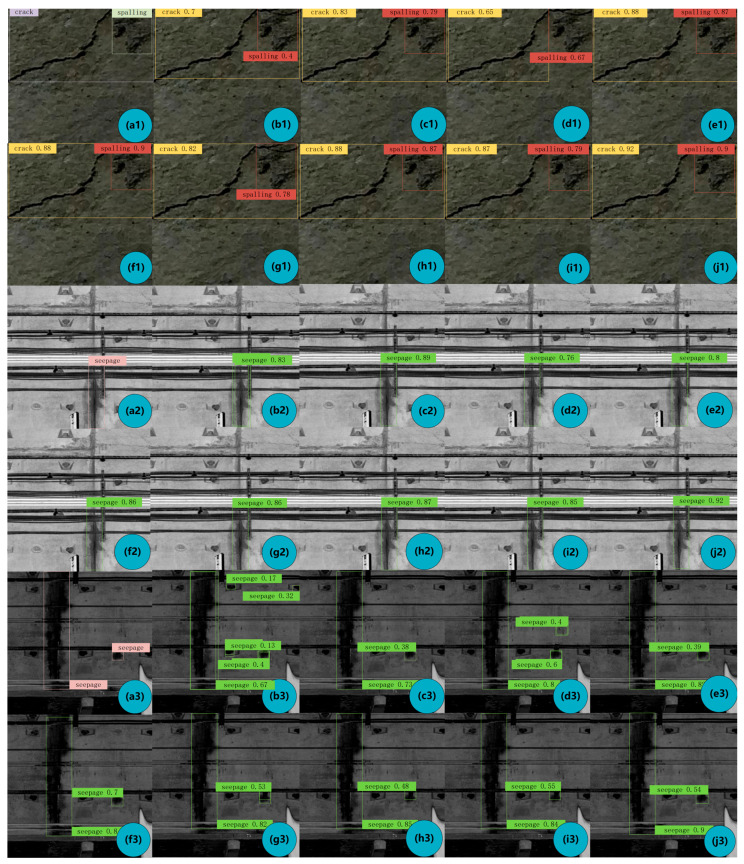

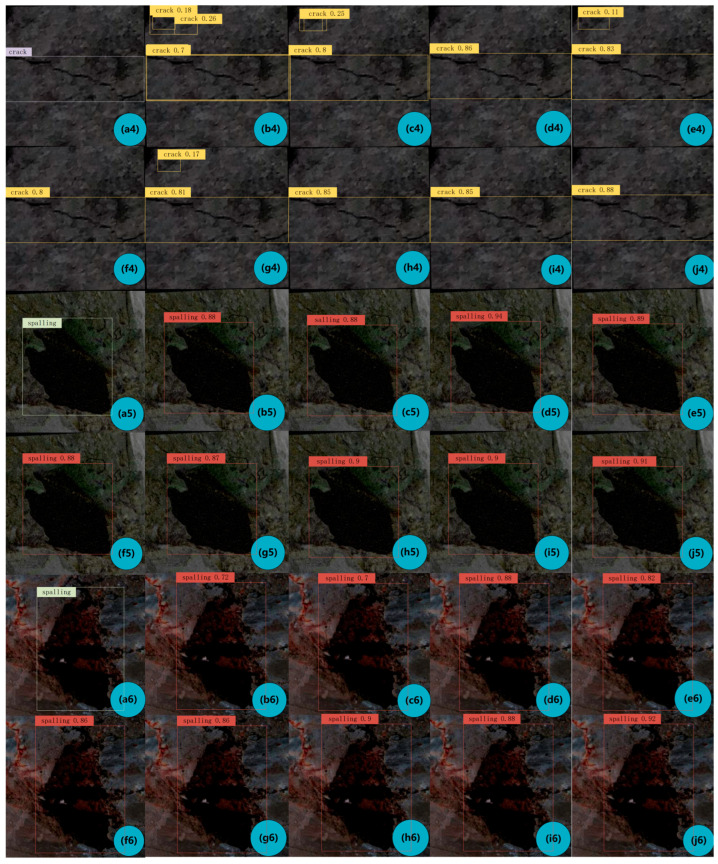
Comparison of detection results of different models (**a**–**j**) for typical tunnel surface defects (**1**–**6**). (**a**) Input. (**b**) Faster R-CNN. (**c**) YOLOv8. (**d**) YOLOv5. (**e**) SSD. (**f**) EfficientDet. (**g**) RetinaNet2. (**h**) CenterNet. (**i**) RT-DETR. (**j**) CAMD-RTDETR.

Figure 11 presents the visualized detection results of different algorithms on the test set. It can be observed that conventional one-stage and two-stage networks often suffer from bounding-box localization drift or relatively low confidence scores when dealing with the complex textures of tunnel surfaces. In particular, for slender crack defects, although RT-DETR shows some improvement, there is still room for enhancement in terms of boundary regression accuracy. In contrast, the proposed CAMD-RTDETR model significantly improves the ability to capture defect features through the incorporation of multi-scale feature fusion and attention mechanisms. The results demonstrate that this method not only achieves the highest bounding-box fitting accuracy but also delivers the best visual detection performance across multiple defect categories.

**Figure 12:**
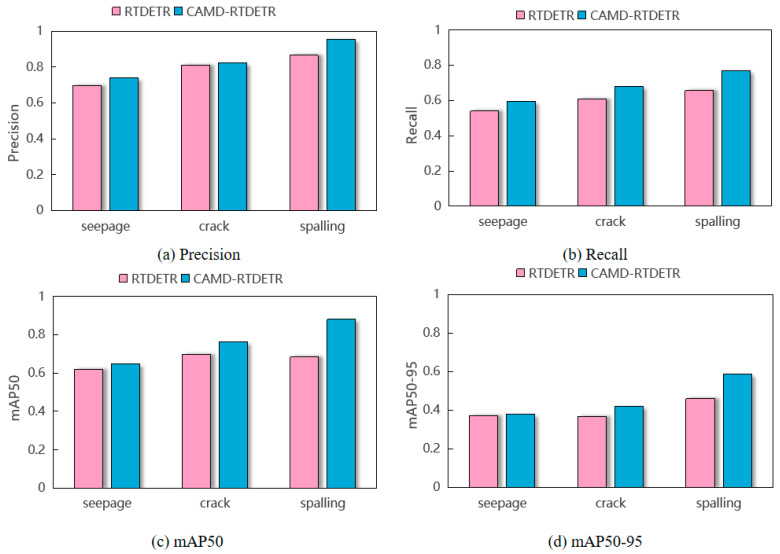
Performance Comparison between RT-DETR and CAMD-RTDETR on Different Defect Types. (**a**) Precision; (**b**) Recall; (**c**) mAP50; (**d**) mAP50-95.

To further analyze the detection performance of the model on different types of tunnel defects, the proposed CAMD-RTDETR was comparatively evaluated against the baseline RT-DETR on three defect categories, namely seepage, cracks, and spalling, as shown in Figure 12. From the perspectives of Precision, Recall, mAP50, and mAP50-95, the results can intuitively reflect the performance differences and advantages of the proposed model under different defect characteristics.

As can be seen from the figure, in terms of Precision, CAMD-RTDETR achieves higher accuracy than RT-DETR across all three defect categories, with the most pronounced improvement observed in spalling detection, indicating that the proposed modules enhance the model’s capability to extract critical defect features. In terms of Recall, the proposed model also shows clear improvements for all three defect categories, demonstrating a stronger ability to detect small targets and low-contrast defects. Regarding mAP50 and mAP50-95, the proposed model exhibits the most significant improvement for the spalling category, with mAP50-95 approaching 0.6, representing a substantial increase over the baseline model. This result highlights its superior detection performance in complex scenarios with significant scale variation.

To further verify the applicability of CAMD-RTDETR in edge-side inspection scenarios, TensorRT-based inference tests were conducted on an NVIDIA edge computing platform. As shown in Table 8, the model achieves 90.1757 FPS with a TRT latency of 11.2739 ms at an input resolution of 416 × 416. When the complete folder-level processing pipeline is considered, including image loading, preprocessing, TensorRT inference, and postprocessing, the model achieves 41.7177 FPS with an average processing latency of 23.9706 ms. At input resolutions of 512 × 512 and 640 × 640, the TRT inference speeds are 78.4775 FPS and 55.1940 FPS, respectively. These results show that the proposed method can maintain practical inference efficiency on edge hardware. It should also be noted that higher input resolutions increase computational cost and reduce inference speed, while lower input resolutions provide faster processing but may affect detection accuracy. Therefore, the input resolution can be selected according to the accuracy–speed requirements of practical vehicle-mounted tunnel inspection tasks.

### 4.4. Model Performance Evaluation Results

**Figure 13:**
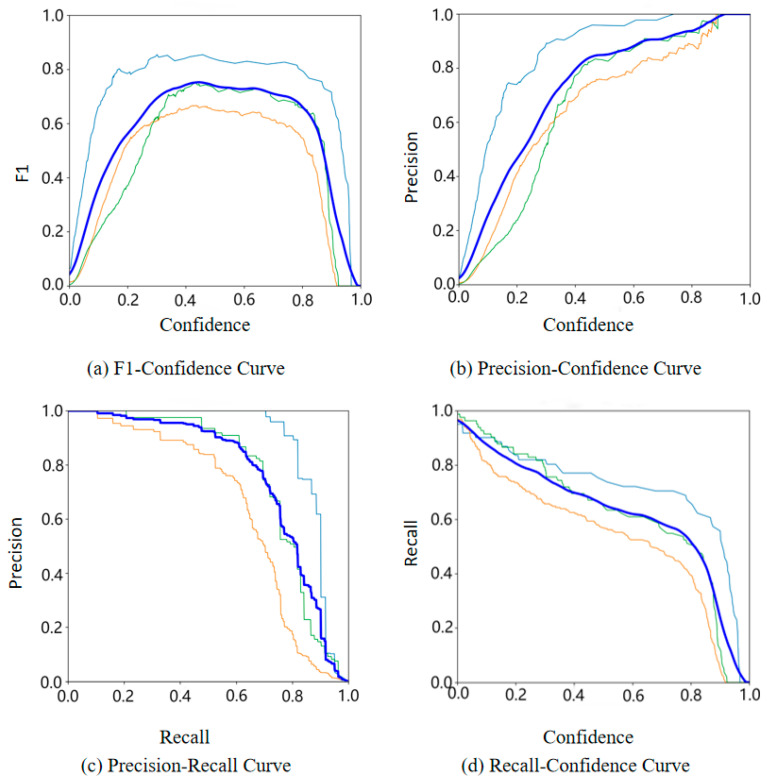
Multi-Metric Performance Curves Based on Confidence. The curves in different colors correspond to the three defect categories—spalling, seepage, and crack—and are used to characterize the detection performance of the classifier under different confidence thresholds. Specifically, the light blue, orange, and green curves represent the performance of each individual category, whereas the bold dark blue curve indicates the overall performance across all categories.

To comprehensively evaluate the detection performance of the proposed model from multiple perspectives, F1–Confidence, Precision–Confidence, Precision–Recall, and Recall–Confidence curves were plotted, as shown in Figure 13. These metrics provide an intuitive visualization of the model’s precision, recall, and their trade-off under different confidence thresholds, thereby facilitating the assessment of the stability and overall performance of the proposed method.

#### 4.4.1. Figure 13a: F1–Confidence Curve

As shown in Figure 13a, the F1 score of the model generally exhibits a trend of first increasing, then remaining stable, and finally decreasing as the confidence threshold changes. In the low-confidence range, a large number of predicted boxes are retained. Although this leads to a relatively high recall, false detections are also more pronounced, resulting in a relatively low F1 score. As the threshold gradually increases, low-quality predictions are effectively suppressed, and the balance between precision and recall is improved. Consequently, the F1 score increases rapidly and remains at a high level within a certain threshold range. When the threshold further increases, some valid targets are incorrectly filtered out, leading to a decrease in recall and, accordingly, a decline in the F1 score. The peak values and variation ranges of the curves differ across defect categories, indicating that the model exhibits different levels of detection sensitivity and stability for different types of defects. Some categories show higher peak values and wider stable intervals, suggesting better detection performance for these defects. Meanwhile, the overall category curve maintains a high level over a relatively wide confidence range, demonstrating that the model can effectively balance precision and recall in multi-category defect detection tasks and possesses strong comprehensive detection capability.

#### 4.4.2. Figure 13b: Precision–Confidence Curve

As shown in Figure 13b, the Precision of the model generally increases as the confidence threshold rises. In the low-confidence range, the model retains a relatively large number of candidate prediction boxes. Although this enables more potential targets to be covered, a certain proportion of low-quality predictions is also included, resulting in more false detections and a relatively low Precision. As the threshold gradually increases, low-confidence erroneous predictions are progressively filtered out, and the accuracy of the remaining detection results is improved. Therefore, Precision continues to increase and tends to stabilize in the high-threshold range. The variation ranges of the curves corresponding to different defect categories differ to some extent, indicating that the model’s discriminative capability is not entirely consistent across different types of defects. Some categories can maintain high Precision even at relatively low thresholds, suggesting that the model identifies these defects more accurately. Meanwhile, the overall category curve exhibits a steady upward trend, demonstrating that the model has good prediction reliability and false-positive suppression capability in the multi-category tunnel defect detection task.

#### 4.4.3. Figure 13c: Precision–Recall Curve

As shown in Figure 13c, the Precision–Recall curve of the model is generally located in a high-performance region, indicating that the model can maintain favorable detection accuracy while ensuring a high recall rate, thereby demonstrating strong overall detection performance. At the low-recall stage, the model outputs relatively conservative predictions, and Precision remains at a high level. As Recall gradually increases, the model needs to cover more ground-truth targets, inevitably introducing some false detections and resulting in a certain decline in Precision. This trend indicates that the model achieves a reasonable trade-off between Precision and Recall. The positions and declining trends of the curves differ among defect categories, suggesting variations in detection difficulty and model adaptability for different types of defects. Some category curves are closer to the upper-right corner, indicating better detection performance for those defect types. Meanwhile, the overall category curve maintains a favorable envelope shape, demonstrating that the model possesses strong comprehensive recognition capability and good generalization performance in the joint detection of multiple tunnel defect categories.

#### 4.4.4. Figure 13d: Recall–Confidence Curve

As shown in Figure 13d, the Recall of the model gradually decreases as the confidence threshold increases. In the low-confidence range, the model retains more prediction results and is therefore able to detect more true targets, resulting in a relatively high Recall. As the threshold further increases, some valid targets with relatively low confidence scores are filtered out, leading to an increase in missed detections and a corresponding decline in Recall. When the threshold enters a higher range, the downward trend of Recall becomes more pronounced, indicating that an excessively high threshold weakens the model’s ability to cover true defect targets. The decay rates of the curves differ among defect categories, suggesting that the detection stability of the model is not completely consistent across different defect types. Some categories maintain a high Recall over a relatively wide threshold range, indicating that the model has strong target-capturing capability for these defects. Meanwhile, the overall category curve still maintains a relatively stable Recall level within the medium-confidence range, demonstrating that the model retains good target detection capability while suppressing false detections.

Based on the four evaluation curves, the proposed model demonstrates favorable detection stability and overall performance under different confidence thresholds. The differences among defect categories in these metric curves indicate that the model exhibits varying recognition capabilities for different types of defects. Specifically, it performs better for certain categories, while there remains room for further improvement for defect types with strong interference from complex textures or blurred boundaries. Overall, the model achieves a good balance among Precision, Recall, and their trade-off in the multi-category tunnel defect detection task, indicating promising potential for engineering applications.

### 4.5. Failure Case and Limitation Analysis

**Figure 14:**
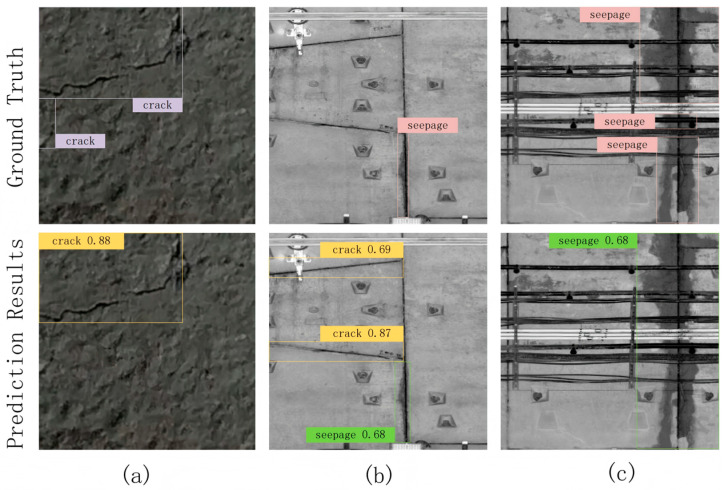
Typical failure cases of the proposed method. (**a**) Partial missed detection of tiny crack targets. (**b**) False detection caused by complex background interference. (**c**) Inaccurate localization of seepage defects.

To further analyze the detection boundaries of the proposed model under complex scenarios, three typical failure cases were selected for visual demonstration, as shown in Figure 14. It can be observed that, in scenarios involving weak-texture and small-scale cracks, the model may still produce missed detections due to insufficient effective feature responses. Under conditions of complex background textures or strong structural interference, certain local regions are prone to being misidentified as defect targets. For seepage regions with irregular shapes and blurred boundaries, the model still exhibits some deviations in characterizing the target extent. These results indicate that, although the proposed CAMD-RTDETR model achieves favorable overall performance, there remains room for further improvement in small-target recognition, background suppression, and boundary localization under extremely complex working conditions.

Although CAMD-RTDETR uses rectangular bounding boxes to detect cracks, seepage, and spalling, the detection results can still provide useful information for practical tunnel inspection workflows. Specifically, the model can be used as a front-end rapid screening tool to automatically identify the defect category and approximate spatial location in tunnel images. The detected bounding boxes can guide inspectors to suspicious regions and reduce the workload of manual image review. In practical maintenance workflows, these detection results can be further combined with manual verification, fine-grained segmentation, geometric measurement, and severity assessment modules to obtain quantitative indicators such as defect area, crack length, crack width, seepage extent, and damage severity.

## 5. Conclusions

Based on the RT-DETR framework, this paper proposes a real-time tunnel defect detection method that balances detection accuracy and inference efficiency for typical defects such as tunnel cracks, water seepage, and spalling. Focusing on key challenges in complex tunnel scenarios, including missed detection of small-scale defects, difficulty in unified representation of multi-scale targets, and insufficient detection stability under complex backgrounds, the proposed method is constructed from three aspects: enhanced feature representation, multi-scale contextual modeling, and stable optimization in the decoding stage. This enables the model to more effectively identify, localize, and classify defect targets at different scales.

Experimental results demonstrate that the proposed method achieves favorable overall performance on the self-constructed tunnel defect dataset. The model achieves a Precision of 0.839, an mAP50 of 0.764, and an inference latency of 5.4 ms, corresponding to a processing speed of approximately 185 FPS. Compared with the baseline RT-DETR model, Precision, Recall, mAP50, and mAP50-95 are improved by 6.3%, 13.5%, 14.7%, and 15.8%, respectively. These results indicate that the proposed method not only improves detection accuracy but also enhances detection stability in complex scenarios. Further comparison with seven commonly used detection models shows that the proposed method achieves superior overall performance in terms of both detection accuracy and inference speed, demonstrating a favorable balance between accuracy and efficiency.

From an engineering perspective, CAMD-RTDETR is intended as a front-end rapid screening and localization tool for tunnel inspection. Its bounding-box outputs can identify defect categories and approximate locations, thereby helping inspectors prioritize suspicious regions and reduce the workload of manual image review. However, the proposed method does not directly quantify crack length or width, defect area, severity, or temporal progression and therefore should not be regarded as a standalone tunnel condition assessment or maintenance decision-making system. Quantitative assessment requires further integration with fine-grained segmentation, geometric measurement, severity evaluation, temporal tracking, and manual verification. Future research may further focus on lightweight deployment, multi-scenario data validation, and adaptability optimization under complex working conditions, so as to continuously improve the generalization performance and practical deployment capability of the model.

## Figures and Tables

**Table 1:** Technical differences between the proposed modules and related existing methods.

Proposed Module	Related Existing Modules	Main Focus of Existing Methods	Technical Novelty in This Study
CAFM	SE, CBAM, Non-local, Deformable Attention	SE and CBAM mainly recalibrate channel or spatial responses within a single feature map. Non-local attention focuses on long-range dependency modeling within one feature map. Deformable Attention enhances spatial sampling and position modeling through sparse adaptive sampling.	CAFM establishes bidirectional cross-level interaction between shallow spatial details and deep semantic features. By constructing a channel correlation matrix, it enables semantic guidance and detailed feedback, thereby improving weak crack perception and boundary localization of large-scale defects.
MSFP	SPP, ASPP, FPN, PANet	SPP and ASPP mainly enlarge the receptive field through multi-scale pooling or atrous convolution. FPN and PANet mainly fuse multi-level features through cross-layer top-down or bottom-up pathways.	MSFP builds lightweight multi-scale contextual representations within the same feature layer using parallel average pooling, feature concatenation, channel compression, and residual connection. It enhances the unified modeling of fine cracks and large-area seepage or spalling defects.
DAE	Deformable DETR, DN-DETR, RT-DETR improvements	Deformable DETR emphasizes multi-scale deformable attention. DN-DETR focuses on denoising training and matching stability. Existing RT-DETR improvements mainly focus on efficient encoding and query selection.	DAE jointly introduces channel attention, spatial attention, and multi-scale deformable attention in the decoding stage. It first enhances the saliency of encoded features and then optimizes query representations, improving query matching stability and localization accuracy under complex tunnel backgrounds.

**Table 2:** Comparison of Detection Performance between CAFM and Other Attention Mechanisms.

Structure	Precision	Recall	mAP50	mAP50-95
CAFM	0.856	0.631	0.752	0.453
SE	0.826	0.656	0.723	0.421
CBAM	0.810	0.639	0.725	0.450
Non-local	0.839	0.609	0.704	0.437
Deformable Attention	0.837	0.630	0.720	0.431

**Table 3:** Comparison of Detection Performance between MSFP and Other Multi-Scale Feature Processing Methods.

	Precision	Recall	mAP50	mAP50-95
MSFP	0.784	0.632	0.727	0.469
SPP	0.788	0.620	0.689	0.408
ASPP	0.752	0.617	0.723	0.443
FPN	0.784	0.609	0.69	0.418
PANet	0.732	0.637	0.701	0.414

**Table 4:** Comparison of Detection Performance between DAE and Representative DETR Variants.

	Precision	Recall	mAP50	mAP50-95
DAE	0.825	0.666	0.726	0.447
DAB-DETR	0.859	0.624	0.707	0.406
Conditional-DETR	0.797	0.659	0.703	0.422
DN-DETR	0.832	0.632	0.726	0.449
Two-Stage-Deformable-DETR	0.838	0.618	0.702	0.408

**Table 5:** Ablation Experiments on Detection Performance of CAFM, MSFP, and DAE Modules.

	DAE	MSFP	CAFM	Precision	Recall	mAP50	mAP50-95
Basic				0.789	0.601	0.666	0.399
1	√			0.825	0.666	0.726	0.447
2		√		0.784	0.632	0.727	0.469
3			√	0.856	0.631	0.752	0.453
4	√	√		0.794	0.651	0.708	0.447
5		√	√	0.840	0.607	0.706	0.420
6	√		√	0.825	0.630	0.694	0.433
7	√	√	√	0.839	0.682	0.764	0.462

“√” indicates that the corresponding module is included in the model, whereas a blank cell indicates that it is not included. “Basic” denotes the baseline RT-DETR model without DAE, MSFP, or CAFM.

**Table 6:** Implementation details of baseline models used for comparison.

Model	Version/Implementation	Backbone	Pretrained Weights	Input Size	Epochs	Optimizer	Initial LR	Augmentation	Confidence/IoU Threshold
Faster R-CNN	Faster R-CNN R50-FPN	ResNet-50-FPN	COCO pretrained	640 × 640	500	SGD	0.01	Same training augmentation	0.25/0.50
SSD	SSD	VGG16/MobileNet-based SSD backbone	COCO pretrained	640 × 640	500	SGD	0.01	Same training augmentation	0.25/0.50
YOLOv5	YOLOv5s	CSPDarknet	COCO pretrained	640 × 640	500	SGD	0.01	Mosaic and geometric augmentation	0.25/0.50
YOLOv8	YOLOv8s	CSPDarknet-based C2f backbone	COCO pretrained	640 × 640	500	SGD	0.01	Mosaic and geometric augmentation	0.25/0.50
EfficientDet	EfficientDet-D0	EfficientNet-B0	COCO pretrained	640 × 640	500	SGD	0.01	Same training augmentation	0.25/0.50
RetinaNet	RetinaNet R50-FPN	ResNet-50-FPN	COCO pretrained	640 × 640	500	SGD	0.01	Same training augmentation	0.25/0.50
CenterNet	CenterNet	ResNet-based backbone	COCO pretrained	640 × 640	500	SGD	0.01	Same training augmentation	0.25/0.50
RT-DETR	RT-DETR-L	ResNet/HGNetv2-based backbone	Official pretrained weights	640 × 640	500	SGD	0.01	Same training augmentation	0.25/0.50
CAMD-RTDETR	Proposed model based on RT-DETR-L	RT-DETR-L backbone with CAFM, MSFP, and DAE	RT-DETR-L pretrained weights	640 × 640	500	SGD	0.01	Same training augmentation	0.25/0.50

**Table 7:** Comparison of Complexity and Inference Efficiency among Different Structural Versions.

Models	Params/M	FLOPs/G	Latency@BS1/ms	FPS@BS1	PeakMem@BS1/MB	Latency@BS2/ms	FPS@BS2	PeakMem@BS2/MB	Latency@BS4/ms	FPS@BS4	PeakMem@BS4/MB
RT-DETR	20.08	58.2	16.758	59.67	178.12	24.779	80.712	244.31	42.208	94.76	377.69
RT-DETR+CAFM	21.24	64.5	17.262	57.92	195.50	27.026	74.002	272.63	51.992	76.93	425.82
RT-DETR+MSFP	23.76	24.4	16.759	59.66	276.19	20.861	95.869	342.37	33.643	118.8	475.75
RT-DETR+DAE	20.69	59.2	17.736	57.85	261.37	22.083	90.564	327.56	42.872	93.29	460.93
CAMD-RTDETR	19.30	57.8	15.855	63.06	335.33	23.194	86.227	401.52	40.987	97.59	534.89

**Table 8:** Edge-side inference performance of CAMD-RTDETR under different input resolutions.

Input Size	TRT FPS	TRT Latency/ms	Folder FPS	Folder Latency/ms	mAP50	mAP50-95	Power/W
416 × 416	90.1757	11.2739	41.7177	23.9706	0.7897	0.4700	8.1018
512 × 512	78.4775	13.8374	36.8891	27.1083	0.7376	0.4316	8.6278
640 × 640	55.1940	18.5877	29.8260	33.5278	0.5729	0.2574	9.2377

## Data Availability

The data presented in this study are available on request from the corresponding author. The data are not publicly available due to privacy and engineering project confidentiality restrictions.
